# Decisional Regret in Long-Term Australian Allogeneic Hematopoietic Stem Cell Transplantation Survivors: A Cross-Sectional Survey

**DOI:** 10.1177/10547738231180337

**Published:** 2023-06-16

**Authors:** Gemma McErlean, Caley Tapp, Lisa Brice, Anisha Pradhan, Nicole Gilroy, Masura Kabir, Matt Greenwood, Stephen R Larsen, John Moore, David Gottlieb, Mark Hertzberg, Louisa Brown, Megan Hogg, Gillian Huang, Christopher Ward, Ian Kerridge

**Affiliations:** 1 University of Wollongong, Sydney, NSW, Australia; 2 New South Wales Agency for Clinical Innovation, Sydney, Australia; 3 University of Queensland, Herston, Australia; 4 Queensland Centre for Mental Health Research, Wacol, Australia; 5 Royal North Shore Hospital, Sydney, NSW, Australia; 6 Westmead Hospital, Sydney, NSW, Australia; 7 Westmead Breast Cancer Institute, Sydney, NSW, Australia; 8 University of Sydney, NSW, Australia; 9 Royal Prince Alfred Hospital, Sydney, NSW, Australia; 10 St Vincents Hospital, Sydney, NSW, Australia; 11 Prince of Wales Hospital, Randwick, NSW, Australia; 12 Calvary Mater Hospital, Newcastle, NSW, Australia

**Keywords:** decisional regret, psychosocial, bone marrow transplantation, hematopoietic stem cell transplant, survivorship, cancer, allogeneic transplant

## Abstract

Allogeneic hematopoietic stem cell transplantation (allo-HSCT) is an intensive but effective treatment for malignant and non-malignant diseases. However, long-term survival often comes at a cost, with survivors experiencing chronic morbidity and are at risk of relapse and secondary malignancy. This study aimed to describe decisional regret in a large cohort of Australian long-term allo-HSCT survivors. A cross-sectional survey was conducted with 441 adults in New South Wales, assessing quality of life (QoL), psychological, social, demographic, and clinical variables. Less than 10% of survivors expressed regret, with chronic graft-versus-host disease being the most important clinical factor. Psycho-socioeconomic factors such as depression, lower QoL scores, lower household income, higher treatment burden, and not resuming sex post-HSCT were also associated with regret. Findings highlight the need for valid informed consent and ongoing follow-up and support for allo-HSCT survivors dealing with life post-transplant. Nurses and healthcare professionals play a critical role in addressing decisional regret in these patients.

## Introduction

Progress in allogeneic hematopoietic stem cell transplantation (allo-HSCT) to treat individuals with malignant and non-malignant disease has resulted in dramatic improvements in survival, with up to 75% of HSCT recipients surviving at least 10 years post-transplant (ABMTRR, 2021; Cieri et al., 2021). While this has significantly improved outcomes for many people with life-threatening illness, survival comes at a cost, with many patients experiencing serious and debilitating chronic morbidity post-HSCT and living with the ongoing threat of relapse and secondary malignancy.

Studies of the experience of survivors of cancer and transplantation have demonstrated that, in hindsight, some survivors come to regret the decision to proceed with treatment, particularly those who had a different treatment option open to them and whose quality of life (QoL), psychological and physical well-being, and relationships are diminished following treatment (Leuthold et al., 2021). This feeling of discomfort or distress regarding a previous decision, in this case to proceed with a recommended course of treatment, is termed “decisional regret” (Szproch & Maguire, 2022). While, of course, regretting one’s decision cannot reverse the decision that has already been made, it is important to understand the prevalence and impact of decisional regret because it may be associated with a range of affective symptoms including anger, guilt, and depression, with lower satisfaction with medical care and with a lower QoL (Cusatis et al., 2020). Importantly, if patients express decisional regret post-treatment, this may also suggest that the process of decision-making and consent, and specifically the degree to which patients genuinely understand the possible adverse consequences that may follow treatment, may be suboptimal.

Studies of different populations of patients who have been treated for cancer suggest that between 3% and 60% may experience some form of decisional regret (Nicolai et al., 2016; Szproch & Maguire, 2022). The likelihood that a person will regret their decision to undergo cancer therapy is, unsurprisingly, greater in those who experience poorer physical or psychological health, reduced QoL, and more adverse effects associated with treatment (Becerra Perez et al., 2016; Christie et al., 2015; Flitcroft et al., 2018; Wilson et al., 2017). Patients also consistently appear to be more likely to experience regret where they are unaware of the negative consequences that may arise as a result of treatment (van Tol-Geerdink et al., 2016). Associations have also been found with a number of psychosocial and demographic factors, including relationship status (single), mental health (depression and anxiety), educational level (lower than tertiary level education), ethnicity (non-white), income and financial security (lower socioeconomic status), employment (unemployed), and social support (socially isolated) (Becerra Perez et al., 2016; Szproch & Maguire, 2022).

Few studies have investigated decisional regret in HSCT survivors. With the exception of a single registry study which conducted secondary analysis on 184 patients from 7 U.S. transplant centers, all others have been small single-center studies (Cusatis et al., 2020; Drevdahl & Dorcy, 2012; Khemani et al., 2018; Leuthold et al., 2021; Mosher et al., 2011). These suggest that decisional regret is uncommon post-HSCT, occurring in less than 15% of HSCT survivors, and may be associated with poorer QoL and disease relapse post-transplant. In this study, we aimed to describe the prevalence and determinants of decisional regret in a large cross-sectional cohort of Australian survivors of allo-HSCT.

## Methods

This was part of a large, multicenter study of the experience of survival of HSCT recipients who had undergone an allo-HSCT in one State in Australia, between January 2000 and December 2012. The larger study sought detailed information about the late consequences of HSCT, their relationships to various sociodemographic and transplant variables, and the effects these had on Australian survivors’ experiences and QoL. The data collected were analyzed by topic and published in appropriate target journals (Brice et al., 2017, 2020; Dyer, Brice, Gilroy, et al., 2018; Dyer, Brice, Schifter, et al., 2018; Dyer, Gilroy, et al., 2016; Dyer, Larsen, et al., 2016; Gifford et al., 2016; Lindsay et al., 2016; Smith et al., 2017).

### Research Setting

This study was conducted in New South Wales (NSW), Australia’s most populous state. NSW covers an area of 800,628 km and in 2022 reported a population of 8.13 million. More than 33% of residents live in regional and rural areas (ABS, 2020). At the time of study inception, there were four adult allo-HSCT centers in NSW, all based in metropolitan Sydney and collectively performing approximately 216 HSCTs annually (ABMTRR, 2013).

### Patients and Procedures

The four adult transplant centers’ allo-HSCT databases were searched for eligible participants. To be eligible, a participant had to be at least 18 years old, have undergone an allo-HSCT between January 1, 2000 and December 31, 2012, have survived for at least a year at the time of survey completion, able to read and write in English, and give consent. The research team received names and phone numbers, and potential participants were either phoned or approached at their HSCT Clinic appointment. Consenting participants had the choice of completing the questionnaire themselves or by phone interview. In all, 187 HSCT survivors who had not returned the survey within a month received a second phone call. The survey was self-completed by all the participants and all co-authors had access to primary clinical trial data. The study protocol was approved by the Northern Sydney Local Health District Human Research Ethics Committee (NSLHD HREC Reference: 1207-217M).

## Instruments

Participants were asked to complete seven instruments. These included six validated survey instruments and one purpose designed specifically for the larger study, the Sydney Post BMT Survey (SPBS). The six validated instruments included the Functional Assessment of Cancer Therapy—Bone Marrow Transplant (FACT—BMT Version 4) (Cella et al., 1993; McQuellon et al., 1997), Depression, Anxiety, and Stress Scale (The DASS 21) (Crawford & Henry, 2003; Dahm et al., 2013; Lovibond & Lovibond, 1996), Chronic Graft-versus-host disease (GVHD) (The Chronic GVHD Activity Assessment—Patient Self Report (Form B) (Pavletic et al., 2006), Lee Chronic GVHD Symptom Scale (Lee et al., 2002), Posttraumatic Growth Inventory score (Morris et al., 2013; Tedeschi & Calhoun, 1996), and the Fear of Cancer Recurrence score (Greenberg et al., 1997). (Please note HSCT and BMT [blood and marrow transplant] are synonymous, we chose to use the term HSCT; however, the tools used in the study were titled with BMT.)

The SPBS was created by the research team following a literature review and discussions with HSCT survivors attending HSCT late effects clinics to cover topics not addressed in existing surveys. The survey comprised 402 questions grouped into 20 domains and included questions regarding demographics, medical complications, infections, vaccinations, complementary therapy use, cancer screening, relationship status, income (Australian Dollars [AUD]), and lifestyle factors following allo-HSCT. The questionnaire used tick box responses, short answer questions, and five-step Likert scales measuring attitudes and other factors and took approximately 1 hour to complete. The questionnaire was piloted with six HSCT survivors to assess face and content validity and to check for comprehension. For each consenting participant transplant, data were also collected including dates of diagnosis and transplant, stage/remission status at transplant, transplant conditioning, GVHD prophylaxis, stem cell source, and donor type.

### GVHD Measurement

Two instruments and one stand-alone question were used to measure the prevalence and impact of GVHD: The Chronic GVHD Activity Assessment—Patient Self Report (Form B) and the Lee Chronic GVHD Symptom Scale. The Chronic GVHD Activity Assessment—Patient Self Report Form B (Pavletic et al., 2006) is a 10-item questionnaire which asks patients to report on the severity and intensity (out of 10) in the last 7 days of skin, oral, ocular, and vulvovaginal symptoms as well as perceived global ratings of GVHD. The Lee Chronic GVHD Symptom Scale is a 30-item validated questionnaire for measuring symptoms of cGVHD (Lee et al., 2002). It consists of seven subscales measuring the adverse effects of cGVHD on skin, eyes, mouth, lungs, nutritional status, muscles and joints, vitality, and psychological functioning. Patients rate themselves over the past month using five-step Likert scales. The stand-alone question asked “Have you ever been diagnosed with chronic GVHD? Yes/No.”

### The Posttraumatic Growth Inventory

The Posttraumatic Growth Inventory is a 21-item questionnaire which measures posttraumatic growth experiences in the lives of trauma survivors (Morris et al., 2013; Tedeschi & Calhoun, 1996). It is widely used to assess the positive life changes following traumatic events such as cancer, HIV, rape, disasters, and other crises. Statements including “I developed new interests,” “I know that I can handle difficult situations,” and “I learned a great deal about how wonderful people are” are expressed and the reader is asked to respond using a six-point Likert scale with responses ranging from, “I did not experience this change” to “I experienced this change to a very great degree as a result of my crisis.” In the current sample, the internal consistency was excellent (Cronbach α = .93).

### The DASS 21

The Depression, Anxiety, and Stress Scale (DASS 21) is a 21-item self-report questionnaire designed to measure the severity of a range of symptoms common to both depression and anxiety (Crawford & Henry, 2003; Dahm et al., 2013; Lovibond & Lovibond, 1996). It uses a four-point Likert scale and each question is scored out of three for an overall total score out of 63. A higher score indicates greater severity of symptoms of anxiety or depression. Internal consistency of the DASS 21 was excellent (Cronbach α = .93) in the current sample.

### Decisional Regret Measurement—FACT-BMT Version 4

The Functional Assessment of Cancer Therapy (FACT-BMT) is a 47-item, valid, and reliable questionnaire for measuring QoL in HSCT recipients (McQuellon et al., 1997). It combines two instruments, the FACT-G and a HSCT subscale. The FACT-G measures QoL in cancer patients (Cella et al., 1993). It consists of five subscales measuring physical, functional, social and emotional well-being, and satisfaction with the doctor/patient relationship. The BMT subscale includes 12 items designed to test QoL in HSCT patients. The FACT-BMT plus the BMT subscale provide an overall QoL score. Patients rate themselves over the past 7 days using five-step Likert scales with responses used to calculate overall QoL and subscale well-being scores. In the current sample, the internal consistency of the FACT-BMT was excellent (Cronbach α = .94).

Importantly, the FACT-BMT also includes two unscored questions, one of which is “I regret having a bone marrow transplant,” with responses ranging from not at all (0) to very much (4). This question was used to measure the prevalence and determinants of decisional regret in long-term HSCT survivors.

### Statistical Analysis

Decisional regret, socio-demographics, transplant details, and comorbidities were analyzed using descriptive statistics. Frequencies and percentages were used to summarize categorical responses, while means and standard deviations were used to summarize parametric continuous variables. Associations were examined using chi-square analyses (or Fisher’s exact test where required) and *t*-tests as appropriate. Statistical analysis was performed using STATA (Version 16.1) statistical package (StataCorp, College Station, TX, USA).

## Results

Across the four transplant centers, a total of 1,475 allo-HSCT were performed in the study period. The completed survey was returned by 441 HSCT survivors (66% of total eligible and 76% of those contacted). As shown in Table 1, 57% of the analysis sample was male, with an average age of 52 years (*SD* = 12.61) at the time of the survey. On average, it had been 5.72 years (*SD* = 3.35) since the HSCT. A total of 423 people (4% missing) completed the question about regretting having the HSCT. Of those who responded to this question, 9.2% reported experiencing some level of decisional regret in the past 7 days.

**Table 1.** Characteristics of Analysis Sample (*n* = 423).

Participant characteristic	*n* (%)
Gender	
Male	243 (57%)
Female	180 (43%)
Time since transplant (years), *M* (*SD*)	5.72 (3.35) Range: 1–14 years
Age at time of survey (years), *M* (*SD*)	52.05 (12.61) Range: 19–79 years
Education	
University (some/completed)	129 (41%)
Other^a^	189 (59%)
Occupational status, post-transplant	
Employed	209 (47.39%)
Other^b^	203( 46.03%)
Household income, post-transplant	
Low	145 (36%)
Middle/high	(64%)
Residential location	
Major city	297 (72%)
Regional or remote	116 (28%)
Relationship status	
Married/de facto	333 (80%)
Single/divorced or separated	85 (20%)
Donor type	
Sibling related	238 (57%)
Matched unrelated	152 (36%)
Haploidentical/mismatched	31 (7%)
Conditioning regimen	
Myeloablative	206 (49%)
Reduced intensity	215 (51%)
Diagnosed with chronic GVHD	
Yes	288 (69%)
No	129 (31%)
Chronic GVHD self-reported severity, *M* (*SD*)	3.52 (2.92)
Number parts of body affected by GVHD, *M* (*SD*)	2.16 (2.12)
Number of comorbidities, *M* (*SD*)	2.45 (1.98)

*Note*. GVHD = graft-versus-host disease.

a Some high school, completed high school, trade qualifications/diploma, and some university.

b Casual, homemaker, unemployed, unable to work, and retired.

c Low income $20,000–$39,999; middle income $40,000–$79,999; and high income ≥$80,000.

### Associations with Lifestyle and Demographic Factors

Analyses revealed few statistically significant associations between decisional regret and various demographic, social, and lifestyle factors (Table 2). No demographic variables were associated with decisional regret. Transplant factors such as the number of years since transplant and the type of conditioning received were not associated with decision regret. In terms of socioeconomic variables, people who experienced decisional regret were more likely to have a low income (i.e., reported earning between $20,000 and $39,999 p.a.) post-transplant, compared to those who indicated no post-HSCT regret. People experiencing decisional regret were also less likely to have resumed sexual activity post-HSCT. A greater proportion of those who reported decisional regret attended medical care more than once a month.

**Table 2.** Decisional Regret Associations with HSCT Survivor’s Demographic and Social Variables, Lifestyle and Transplant Factors, and Medical Care.

Participant characteristics	HSCT regret (*n* = 39)	No HSCT regret (*n* = 384)	*p*
Demographic variables			
Gender			
Male	25 (64%)	218 (27%)	.378
Female	14 (36%)	166 (43%)	
Age (years), *M* (*SD*)	50.67 (13.42)	52.19 (12.54)	.473
Postcode			
City-metro	29 (76%)	268 (71%)	.526
Regional or remote	9 (24%)	107 (29%)	
Marital status			
Married or de facto	31 (79%)	302 (80%)	.977
Other^a^	8 (21%)	77 (20%)	
Socioeconomic variables			
Education			
University education	10 (42%)	119 (40%)	.909
No university education	14 (58%)	175 (60%)	
Post-transplant income^b^			
Low income	21 (57%)	124 (34%)	.005
Middle/high income	16 (43%)	246 (66%)	
Occupational status			
Employed	11 (34%)	189 (52%)	.053
Other^c^	21 (66%)	173 (48%)	
Lifestyle factors			
BMI			
Healthy weight	19 (51%)	170 (48%)	.677
Not healthy weight	18 (49%)	186 (52%)	
Travel post-HSCT			
Yes	15 (41%)	210 (55%)	.086
No	22 (59%)	170 (45%)	
Sex post-HSCT			
Yes	19 (53%)	259 (71%)	.024
No	17 (47%)	106 (29%)	
Transplant factors			
Years since transplant			
<2 to 5	25 (68%)	200 (55%)	.155
6+	12 (32%)	161 (45%)	
Years since transplant, *M* (*SD*)	4.97 (2.86)	5.79 (3.39)	.156
Conditioning			
Myeloablative	18 (46%)	188 (49%)	.716
Reduced intensity	21 (54%)	194 (51%)	
Medical care			
Time spent attending medical care			
≤Monthly	22 (58%)	282 (75%)	.025
≥Monthly	16 (42%)	95 (25%)	
Complementary therapy use			
Yes	21 (54%)	204 (53%)	.931
No	18 (46%)	180 (47%)	

*Note.* Fisher’s exact test used where cell size ≤5. BMI = body mass index; HSCT = hematopoietic stem cell transplantation.

a Single, divorced, separated.

b Low income = $20,000–$39,999; middle income = $40,000–$79,999; and high income = $80,000+.

c Casual, homemaker, unemployed, unable to work, and retired.

### Associations with Clinical and Psychological Factors

Results of analyses examining the associations between decisional regret and clinical and psychological factors are reported in Table 3. There were significant associations found between decisional regret and GVHD. Decisional regret significantly increased in HSCT survivors who had previously been diagnosed with chronic GVHD (cGVHD). Those who reported experiencing some degree of decisional regret were also more likely to have severe GVHD (57% reporting moderate or severe GVHD vs. 37% of those with no decision regret; *p* < .001), and have more organ systems affected by GVHD (mean of 3.62 vs. 2.01; *p* < .001).

**Table 3.** Decisional Regret Association with HSCT Survivor’s Clinical and Psychological Factors.

Participant characteristics	HSCT regret (*n* = 39)	No HSCT regret (*n* = 384)	*p*
Clinical factors			
Pretransplant cancer diagnosis			
Yes	4 (11%)	52 (14%)	.803
No	33 (89%)	316 (86%)	
Comorbidities			
1–2 Conditions	15 (38%)	166 (43%)	.137
3+ Conditions	21 (54%)	150 (39%)	
No conditions	3 (8%)	68 (18%)	
GvHD			
Yes	34 (87%)	254 (67%)	**.010**
No	5 (13%)	124 (33%)	
Severity of GVHD^a^			
None/mild	12 (43%)	137 (64%)	**.033**
Moderate/severe	16 (57%)	78 (36%)	
Chronic GvHD self-reported severity, *M* (*SD*)	5.50 (*2.94*)	3.28 (*2.83*)	**<.001**
Number parts of organ systems affected by GvHD, *M* (*SD*)	3.62 (*2.46*)	2.01 (*2.04*)	**<.001**
Secondary cancers, post-HSCT			
Mouth cancer			
Yes	1 (3%)	4 (1%)	.349
No	30 (97%)	344 (99%)	
Skin cancer			
Yes	11 (33%)	78 (22%)	.138
No	22 (67%)	277 (78%)	
Any other cancer			
Yes	5 (16%)	21 (6%)	0.61
No	26 (84%)	307 (94%)	
Psychological factors			
DASS, *M* (*SD*)	45.03 (*33.17*)	24.19 (*20.89*)	**<.001**
Ever diagnosed since HSCT			
Depression			
Yes	16 (47%)	75 (21%)	**.001**
No	18 (53%)	282 (79%)	
Anxiety			
Yes	10 (30%)	68 (19%)	.129
No	23 (70%)	286 (81%)	
Posttraumatic growth score, *M* (*SD*)	51.05 (*19.77*)	49.35 (*21.22*)	.635
FACT-BMT, *M* (*SD*)	84.42 (*22.11*)	108.15 (*20.29*)	**<.001**

*Note.* DASS = Depression, Anxiety, and Stress Scale; FACT-BMT = Functional Assessment of Cancer Therapy—Bone Marrow Transplantation measure; GVHD = graft-versus-host disease; HSCT = hematopoietic stem cell transplantation.

a Question asked only of those who indicated they had experienced GVHD.

Bold text denotes a significant p value.

There were also differences in psychological factors between those who reported decisional regret and those who did not. Those who experienced decisional regret had a higher score on the DASS 21 total score (45.03 vs. 24.19), indicating more severe overall distress and a greater number of psychological symptoms being experienced (*p* < .001). This was also the case for depression diagnoses, with 47% of those experiencing decisional regret reporting they had been diagnosed (vs. 21% of those with no HSCT regret) with depression (*p* = .001). Interestingly, there was no association between decisional regret and anxiety. There was also no significant difference in posttraumatic growth between the decisional regret groups.

Finally, people who reported transplant decisional regret had a lower QoL score as measured by the FACT-BMT, compared to people reporting no post-HSCT regret (84.42 vs. 108.15; *p* < .001).

There were no statistically significant associations between decisional regret and a pre-transplant cancer diagnosis or the number of comorbidities a person reported between the decisional regret groups. There was also no significant association between secondary cancers and decisional regret.

## Discussion

This is the largest study assessing decisional regret in long-term survivors of allo-HSCT, and the first such study to be done in Australia. Our results reveal that less than 10% of HSCT survivors regret having a transplant. This is consistent with previous U.S. surveys which report rates of decisional regret between 1% and 15% (Cusatis et al., 2020; Leuthold et al., 2021; Mosher et al., 2011). In many ways, of course, this is unsurprising given the life-saving nature of transplant and the limited treatments options that are generally open to the vast majority of patients at the time that they are recommended to undergo HSCT.

Of HSCT survivors in our study who reported decisional regret, the single most important clinical factor associated with its occurrence was chronic GVHD. We also found associations between decisional regret and a number of psycho-socioeconomic factors including depression, lower QOL scores, lower household income strata after HSCT, higher treatment burden (continuing to spend more time attending to medical care), and a failure to resume sexual activity post-HSCT all associated with higher rates of regret about the decision to undergo HSCT.

While the association between chronic GvHD and decisional regret, and between the severity and extent of chronic GvHD and decisional regret, is unsurprising given the relationship between GvHD, survival, psychological symptoms, and QoL post-HSCT, and this is the first study to report this in survivors of HSCT (Gooptu & Antin, 2021). The only other study to explore associations between chronic GvHD and decisional regret—Cusatis et al. (2020) found that chronic GvHD did not predict decisional regret. There are a number of possible explanations for the apparent discrepancy between our study and that by Cusatis and colleagues. While Cusatis et al. (2020) assessed decisional regret at 6 and 12 months post-HSCT, participants in our study had survived, on average 5.7 years post-HSCT. This is relevant because survivors who are 12 months or less post-HSCT are more likely to be dealing with acute adverse effects of HSCT and with early complications of HSCT, such as disease relapse, infection, and acute GvHD, than with chronic GvHD. In addition, respondents to our survey experienced higher rates of GVHD (69% vs. 43%), and had lived with it for longer than the participants in the study by Cusatis et al. (2020), meaning, perhaps, that the respondents in our study would have had more time to reflect on the quality of their life and their satisfaction with decisions they had previously made about their health care and their life.

In contrast, like the Cusatis et al.’s (2020) study, we found that survivors who report better QoL (measure by FACT-BMT) report less decisional regret—a finding that makes intuitive sense given the factors that determine QoL (Cusatis et al., 2020).

As noted, we also found an association between decisional regret and the failure to resume sexual activity post-HSCT. This is a unique and important finding, because sexual dysfunction is extremely common post-HSCT; sexual function and intimacy are major determinants of QoL; sexual dysfunction and genital GvHD are often not recognized or are under-recognized in survivors of HSCT; and it is increasingly recognized that attention to sexual health is an important part of survivorship care (Hirsch et al., 2012; Mueller et al., 2013; Schover et al., 2014; Thygesen et al., 2012).

While it is tempting to suggest the fact that few HSCT recipients experience decisional regret means that it is not a real concern, an alternative view is that transplant services should do what they can to ensure that all HSCT recipients remain accepting of their decisions irrespective of their experiences of survival and should reflect on what can be done to improve consent processes and post-HSCT care (Cusatis et al., 2022). Innumerable strategies may be adopted to improve the validity of consent and help HSCT recipients understand, plan for and manage the uncertainty and complexity that characterizes transplantation. Web-based, culturally sensitive, patient education resources; e-consent platforms; use of survivorship narratives; and/or involvement of HSCT survivors in educational programs for HSCT recipients and their families and multidisciplinary HSCT information seminars may all improve the degree to which HSCT recipients understand what lies in front of them and help them feel part of a process of shared decision-making—even where there are limited options available or where other care pathways lead inevitably to terminal care. These educational strategies could combine multimodal, culturally sensitive educational and assessment interventions (Cusatis et al., 2022; Syrjala et al., 2021) that address all the issues that collectively influence the quality of survival of HSCT survivors and the likelihood that they may experience post-HSCT regret, including social relationships and sexual function, as well as strategies that may assist HSCT recipients deal with an uncertain future. Interventions that have already been developed, like that developed by and evaluated in a randomized controlled trial (RCT) by Prostate Cancer Canada—show that a multimodal educational portal may reduce decisional regret when compared to simple information provision and may provide some guidance (Feldman-Stewart & Siemens, 2015). While similar resources would need to be tailored to the specific needs of HSCT recipients, it is likely that this could increase the understanding of the vulnerability and unpredictability of life post-transplant of patients, carers, and clinicians, and enable more fully informed decision-making and consent.

While this study has many strengths—the large sample size and response rate (76%) making it likely that the results represent an accurate account of the experience of contemporary long-term survivors of allo-HSCT in Australia—there are a number of limitations that may impact the generalizability of these results to allo-HSCT survivors in other countries. First, despite the high response rate, participation was incomplete, resulting in the possible participation bias. Second, this was a cross-sectional study and included only those alive at study recruitment, thereby excluding those who may have died because of post-transplant complications, relapse, and/or multi-morbidity, and therefore those who may have experienced more decisional regret. Also, using a single item (in the FACT-BMT questionnaire) to operationalize decision regret in HSCT and asking this at a single point in time arguably underestimates the likelihood of decisional regret and does not allow for deeper exploration of the meaning and impact of decisional regret post-HSCT. An additional limitation is that respondents in this study were disproportionately white/Caucasians (86.9%), thus limiting the ability to generalize to other ethnic groups. This is significant particularly as Australia is a diverse nation and culturally, ethnically, and linguistically diverse and indigenous minority populations (Aboriginal and Torres Strait Islanders), who may have differing conceptions of health and wellness and experience systemic barriers and inequities in access to care. This is relevant particularly in regard to our understandings of consent and decision-making around HSCT as international research has consistently found that cultural worldviews, education, ethnicity, and social context all shape decision-making preferences of people with cancer (Yennurajalingam et al., 2018).

## Conclusion

This study has described the prevalence and determinants of decisional regret in an Australian cohort of HSCT survivors. We found very few people expressed decisional regret; however for those that did, as other studies have found, lower QoL, poorer physical or psychological health, and more adverse effects/high treatment burden after transplant were associated with decisional regret. In addition, however, we found that decisional regret was strongly associated with the experience of chronic GvHD post-HSCT and with a failure to resume sexual activity post-HSCT. The results of this study are of particular significance for nurses, as the health professionals who have the closest and most enduring contact with HSCT recipients, as it highlights previously unidentified deleterious effects of GVHD and the fact that some HSCT recipients carry with them the terrible sense that they should not have agreed to undergo transplant. The data also add to the literature on the importance of sexual function and cancer survivorship, and the notion that all HSCT recipients need ongoing access to long-term follow-up and survivorship support, education, and evidence-based care to help them deal with life post-transplant.

## Implications for Nursing Practice and Nurse-led Research

HSCT and cancer nurses play a pivotal role in facilitating education and decision-making, and in advocating for patients’ autonomy and ensuring that their preferences and values are considered in the provision of their care. Accordingly, nurses need to be aware of the potential for, and risk factors of, decisional regret in the years post-HSCT, and be prepared to provide support and counseling for those in need. At each contact point, nurses should actively encourage and enable patients and their loved ones to obtain comprehensive, accurate information about their likely course of recovery and their healthcare options. They should also ensure that any misconceptions or unrealistic expectations are addressed. Furthermore, HSCT nurses and other healthcare providers should work together to assist patients and loved ones to build a supportive network to help them cope with the challenges of survival post-HSCT. Nurses are in a unique position to lead research on developing, implementing, and evaluating supportive care research (including things like production of survivor videos, education resources, and, culturally sensitive educational materials), and its impact on decisional regret and long-term satisfaction with care.
